# Central nervous system involvement in childhood acute lymphoblastic leukemia: challenges and solutions

**DOI:** 10.1038/s41375-022-01714-x

**Published:** 2022-10-20

**Authors:** Maria Thastrup, Alasdair Duguid, Christian Mirian, Kjeld Schmiegelow, Christina Halsey

**Affiliations:** 1 Department of Pediatrics and Adolescent Medicine, Rigshospitalet, University of Copenhagen, Copenhagen, Denmark; 2 Centre for Regenerative Medicine, University of Edinburgh, Edinburgh, UK; 3 Novo Nordisk Foundation Center for Protein Research, Proteomics Program, Faculty of Health and Medical Sciences, University of Copenhagen, Copenhagen, Denmark; 4 Institute of Clinical Medicine, Faculty of Health and Medical Sciences, University of Copenhagen, Copenhagen, Denmark; 5 Wolfson Wohl Cancer Research Centre, School of Cancer Sciences, College of Medical Veterinary and Life Sciences, University of Glasgow, Glasgow, UK

**Keywords:** Acute lymphocytic leukaemia, Translational research, Metastasis, Paediatrics

## Abstract

Delivery of effective anti-leukemic agents to the central nervous system (CNS) is considered essential for cure of childhood acute lymphoblastic leukemia. Current CNS-directed therapy comprises systemic therapy with good CNS-penetration accompanied by repeated intrathecal treatments up to 26 times over 2–3 years. This approach prevents most CNS relapses, but is associated with significant short and long term neurotoxicity. Despite this burdensome therapy, there have been no new drugs licensed for CNS-leukemia since the 1960s, when very limited anti-leukemic agents were available and there was no mechanistic understanding of leukemia survival in the CNS. Another major barrier to improved treatment is that we cannot accurately identify children at risk of CNS relapse, or monitor response to treatment, due to a lack of sensitive biomarkers. A paradigm shift in treating the CNS is needed. The challenges are clear – we cannot measure CNS leukemic load, trials have been unable to establish the most effective CNS treatment regimens, and non-toxic approaches for relapsed, refractory, or intolerant patients are lacking. In this review we discuss these challenges and highlight research advances aiming to provide solutions. Unlocking the potential of risk-adapted non-toxic CNS-directed therapy requires; (1) discovery of robust diagnostic, prognostic and response biomarkers for CNS-leukemia, (2) identification of novel therapeutic targets combined with associated investment in drug development and early-phase trials and (3) engineering of immunotherapies to overcome the unique challenges of the CNS microenvironment. Fortunately, research into CNS-ALL is now making progress in addressing these unmet needs: biomarkers, such as CSF-flow cytometry, are now being tested in prospective trials, novel drugs are being tested in Phase I/II trials, and immunotherapies are increasingly available to patients with CNS relapses. The future is hopeful for improved management of the CNS over the next decade.

## Introduction

Childhood acute lymphoblastic leukemia (ALL) is a curable disease with more than 90% of children achieving long term survival [1, 2]. This has led to a shift in focus from intensifying treatment to achieving cure with fewer side-effects. Progress is being made by use of minimal residual disease (MRD) monitoring to adjust treatment intensity according to clinical response, and more recently the introduction of targeted immunotherapies. However, major challenges remain. One particularly problematic area is how best to prevent and/or treat leukemic relapse involving the central nervous system (CNS). Detection of leukemic blasts in the CNS by cytology is commoner in patients with higher white blood cell count at diagnosis, T-lineage ALL, and high risk cytogenetics, but early studies in the 1960s and 1970s established that giving CNS directed therapy to all patients, including those with negative CSF-cytology, is essential for cure [3, 4]. Initial protocols used craniospinal irradiation as CNS-directed treatment. Unfortunately, this caused high rates of neurocognitive impairment [5, 6] and secondary CNS malignancies [7], leading to a shift towards chemotherapy approaches [8]. However, significant toxicity can still occur. The balance between adequate treatment to prevent relapse, whilst minimizing chemotherapy exposure to reduce adverse effects, is especially important when it comes to a child’s developing brain [9]. Here we review the current clinical challenges in CNS-ALL and discuss possible solutions.

## Challenge 1 – Inability to accurately measure CNS involvement

CNS staging is usually performed by counting white blood cells in the cerebrospinal fluid (CSF) along with microscopy of a cyto-centrifuged CSF sample to morphologically identify leukemic blasts. This is used to assign patients to CNS1, CNS2 or CNS3 status (Table 1). CNS3 status is also given to patients with clinical or radiological evidence of CNS-leukemia, irrespective of CSF findings e.g. cranial nerve palsies, or other neurological symptoms that mostly, but not always, are associated with CNS-imaging findings.

**Table 1:** CNS status definitions [71, 129–131].

	CSF cytospin findings		
CNS status	WBCs/μL	RBCs/μL	Leukemic blasts
CNS1	≤5	<10	Absent
CNS2	≤5	<10	Present
CNS3	>5	<10	Present
TLP+	N/A	≥10	Present
TLP−	N/A	≥10	Absent

*CNS* central nervous system, *CSF* cerebrospinal fluid, *N/A* not applicable, *RBCs* red blood cells, *TLP* traumatic lumbar puncture, *WBCs* white blood cells.

Although this method of CNS-staging has been used for decades, it is debatable how clinically or biologically meaningful it is. Several observations challenge whether cytology accurately reflects the amount of leukemic infiltration in the CNS and the likelihood of CNS relapse. Firstly, rates of CNS2 status vary widely between individual centers, and between trial groups, as does their prognostic relevance (Table 2) – suggesting that this is an analytical, rather than a clinical, difference. Indeed, cytospin-based cytology has been shown to have low sensitivity, poor specificity, and low reproducibility between laboratories [10, 11]. Leukocytes in CSF rapidly decay ex vivo [12–14], and if cytospin samples are not stabilized or processed immediately, this may lead to underestimation of CNS leukemia. Furthermore, microscopy-based analysis has a low sensitivity for detection of rare events [15], and discrimination of leukemic cells and normal/reactive T-lymphocytes in cytospins may be difficult and can lead to false-positives [10]. Further doubt on the sensitivity of CNS cytology comes from clinical observations – only 2–5% of children are classified as CNS3 at initial diagnosis, but up to 75% used to relapse within a few weeks to months prior to institution of universal CNS-directed therapy [3] suggesting that leukemia is present in the CNS compartment from disease outset in most patients. This is also supported by animal models [16] and by evidence that patients often have significant CNS infiltration on post-mortem brain biopsies despite lack of cells in the CSF [17]. Moreover, even in patients with CNS3 status, the cells in CSF usually become undetectable after only 1–3 intrathecal treatments despite clinical knowledge that prolonged treatment is required to reduce risk of CNS relapse [4]. Thus, CSF cytology is unable to differentiate good from poor treatment responders. Finally, most CNS relapses occur in children who were CNS1 – therefore, the prognostic value of CNS1–3 status is quite poor. It is clear that better diagnostic, response and prognostic biomarkers are needed for CNS-ALL.

**Table 2:** CNS status at diagnosis, use of prophylactic cranial radiotherapy and outcomes in selected childhood ALL trials [8, 22, 23, 34, 37, 132–145].

			CNS status at diagnosis, %						5-year cumulative incidence of CNS relapse, %		
Study	Recruitment period	Patients, *n*	CNS1	CNS2	CNS3	TLP+	CNS positive [definition where available]	Indication for prophylactic cranial irradiation	Isolated	Combined	5-year EFS, %
AIEOP-BFM ALL2000 [34, 132, 133]	2000–2006	3720	n/a	n/a	n/a	n/a	2.5	MRD levels ≥10 − 3 at day 78, no CR at day 33, t(4;11) translocation, and poor prednisolone response	1.5	1.2	82.2
CCG 1991^§^ [134]	2000–2005	1769	92.1	3.8	2.0	2.1		No	1.8	0.7	90.7
COALL 07–03 [135]	2003–2010	763	n/a	n/a	n/a	n/a	2.6 [non-traumatic LP with leukocytes >5/uL in CSF]	B-precursor ALL and an initial WBC count of >200/nL or 100 to 200/nL and >1 × 109/L leukemic blasts in the peripheral blood at day 8, and patients with T-ALL with a WBC count of ≥50/nL	1.4	2.1	84.3
COG AALL 0232 [37]	2004–2011	2914	85.9	14.1	n/a	n/a		BM D15 M2 or M3, or D29 MRD > 0.1%	n/a	n/a	75.3
COG AALL 0331 [23, 136]	2005–2010	5299	91.0	8.0	1.0	n/a		No	2.0^‡^	0.8^‡^	89.0^‡^
DCOG ALL9 [137]	1997–2004	859	n/a	n/a	n/a	n/a	2.4 [CNS3 (>5 cells/uL + blasts with <15 RBC/uL) or CT/MRI features]	No	2.6	0.8	83.3
DCOG ALL10 [138]	2004–2012	778	42.4	42.2	1.0	10.3		Patients aged >3 years on non-transplant protocol with TP1 MRD level of ≥5 × 10 − 4 or unknown and TP2 MRD level of ≥5 × 10 − 4, MLL-AF4 rearrangements, poor prednisone response, or no CR day 33	1.4	0.9	88.7
DFCI 00–01 [22]	2000–2004	487	84.2	12.3	3.5	n/a		WBC ≥ 50,000/μL, non-B-precursor phenotype, or prescence of mediastinal mass	1.5	2.7	80.0
JACLS ALL-02 [139]	2002–2008	1047	90.6	2.6	2.2	0.6		No	0.8^†^	0.6^†^	85.4^†^
NOPHO ALL2000 [140, 141]	2002–2007	1020	97.1*	n/a	2.9	n/a		Any age and WBC ≥ 200 × 109 l–1, BM D29 M3, t(9;22)(q34;q11), t(4;11)(q21;q23), or low hypodiploidy. And ≥5 years WBC 100—200 × 109 l–1 or T-ALL and mediastinal mass	2.7	2.1	79.4
SJCRH total thearpy 15 [8]	2000–2007	498	72.1	20.5	1.8	5.6		No	2.7	0.8	85.6
SJCRH total thearpy 16 [142]	2007–2017	598	57.4	33.4	3.5	5.7		No	1.2	0.3	88.2
TCCSG L99-15 [143]	1999–2003	754	n/a	n/a	n/a	n/a	2.9 [CNS-1s, CNS-2, CNS-3, TPL+ blasts]	WBC ≥ 100	1.6†	1.5^†^	78.2^†^
UKALL2003 [144, 145]	2003–2011	3113	93.8	5.1	1.1	n/a	n/a	CNS3 until August 2009	1.9	1.1	87.2

*ALL* acute lymphoblastic leukemia, *BM* bone marrow, *CNS* central nervous system, *CR* complete remission, *CSF* cerebrospinal fluid, *CT* computed tomography, *EFS* event free survival, *M2* bone marrow has 5–25% leukemic blasts, *M3* bone marrow has >25% leukemic blasts, *MRD* minimal residual disease, *MRI* magnetic resonance imaging, *RBC* red blood cells, *TLP+* traumatic lumbar puncture with blasts present on cytospin, *WBC* white blood cells.

*AIEOP-BFM* l’Associazione Italiana di Ematologia e Oncologia Pediatrica – Berlin Frankfurt Münter, *CCG* Children’s Cancer Group, *COALL* Cooperative Acute Lymphoblastic Leukemia Study Group, *COG ALL* Children’s Oncology Group Acute Lymphoblastic Leukemia, *DCOG* Dutch Childhood Oncology Group, *DFCI* Dana-Farber Cancer Institute. *JACLS* Japan Childhood Leukemia Study Group. *NOPHO* Nordic Society of Paediatric Haematology and Oncology. *SJCRH* St. Jude Children’s Research Hospital. *TCCSG* Tokyo Children’s Cancer Study Group. *UKALL* United Kingdom Medical Research Council Acute Lymphoblastic Leukaemia.

*Combined CNS1 and CNS2.

^†^4-year EFS.

^‡^6-year EFS.

^§^Derived from methotrexate randomization data.

The timing of when to perform the diagnostic lumbar puncture is also controversial. Some reports suggest that delaying the first lumbar puncture is associated with lower traumatic lumbar puncture (TLP) rates and less CNS relapse [18, 19]. However, no randomized comparisons have been performed, and some practitioners are concerned that delayed LP may interfere with CNS-staging. This is particularly important for protocols that use CNS status to stratify the CNS and systemic therapy intensity (see below). More sensitive biomarkers are required before this question can be properly answered [20].

In keeping with the large variability in CNS2/3 rates across different protocols, and conflicting data on CNS2 status as an independent prognostic factor (Table 2), it is unsurprising that different trial groups have different approaches to using CNS-staging to allocate treatment. In some protocols patients with CNS3 status are assigned to high-risk treatment arms (or at least excluded from low-risk arms), whilst in others they receive additional intrathecal therapies in induction, but no change to their overall risk-group allocation (Table 3). For CNS2 status there is even more controversy, with trials using additional intrathecal treatments for CNS2 patients reporting that CNS2 has no impact on prognosis [21, 22], in contrast to trials which did not escalate therapy [23]. Traumatic lumbar punctures with blasts (TLP+) are associated with particularly poor outcomes and generally considered to require additional CNS-directed therapy [24]. The reason why TLP+ has such a poor outcome is currently unknown, it may reflect a correlation with high-risk disease characteristics, rather than the prevalent, but unlikely, hypothesis that it introduces blasts from the periphery into an otherwise leukemia-free CSF.

**Table 3:** Current CNS-directed therapy approaches taken by selected study groups.

Study group	CNS2 definition	CNS2 management	CNS3 management	TLP + management	Delayed LP for high WCC	Other factors
Children’s Oncology Group ALL committee	Cell count + cytospin	Additional intrathecal therapy	Higher risk treatment arm + additional intrathecal therapy	Individualized	No	B but not T cell ALL CNS2 patients receive additional Its during induction
St Judes Total Therapy Program	Cell count + cytospin (confirmed by Tdt staining)	Additional intrathecal therapy	Additional intrathecal therapy	Additional intrathercal therapy	Optional	Depending on WBC >100,000, T-phenotype, TCF3-PBX1, BCR-ABL1, KMT2A rearrangement, hypodiploidy
Dana-Farber Cancer Institue ALL Consortium	Cell count + cytospin	Additional intrathecal therapy	Higher risk treatment arm + cranial irradiation + additional intrathecal therapy	Assigned to CNS1/2/3 using Steinherz/Bleyer formula	No	No
Japanese Pediatric Leukemia/Lymphoma Study Group	Cell count + cytospin	Additional intrathecal therapy	Higher risk treatment arm	Assigned to CNS1/2/3 using Steinherz/Bleyer formula	Optional, but before day 8	No
l’Associazione Italiana di Ematologia e Oncologia Pediatrica – Berlin Frankfurt Münster	Cell count + cytospin	As per CNS1	Additional intrathecal therapy	Assigned to CNS1/2/3 using study protocol algorithm	Optional, but before day 3	No
ALLTogether Consortium	Cell count + cytospin (confirmed by flow cytometry)	Additional intrathecal therapy	Higher risk treatment arm + additional intrathecal therapy	Higher risk treatment arm + additional intrathecal therapy depending on white cell count	Optional delay until WBC < 50×109/L	No
ALL Intercontinental Berlin-Frankfurt-Münster Study Group	Cell count + cytospin	Additional intrathecal therapy	Cranial irradiation + additional intrathecal therapy	Additional intrathercal therapy	No	No

Most importantly, the inability to accurately quantify CNS leukemic load and response to treatment means that intensive CNS-directed therapy is given to all patients, even in those whose disease is judged to be at “ultra-low” risk of relapse based on karyotype and bone-marrow (BM) MRD quantification. Sensitive response biomarkers capable of accurately quantifying CSF MRD and thus the dynamics of CSF clearance are clearly needed.

## Challenge 2 – What is the most effective CNS-directed therapy regimen for newly diagnosed patients?

### Overview

All modern ALL protocols employ a combination of systemic therapy and intrathecal (IT) therapy against CNS leukemia, with some trial groups also using radiotherapy for selected high-risk groups. This upfront CNS therapy is sometimes called “CNS prophylaxis”, which reflects the aim to prevent CNS relapse. However, it can be wrongly misconstrued as meaning it is preventing dissemination of leukemia to the CNS, although CNS leukemia is likely to be present at the time of diagnosis. Thus, “CNS-directed therapy” is a more appropriate term than “CNS prophylaxis”. The current CNS-directed therapy approaches taken by selected study groups are summarized in Table 3.

The main agents with significant CNS activity are intrathecal and systemic glucocorticoids (not least dexamethasone), intrathecal or intravenous (high-dose) methotrexate and cytarabine, and asparaginase. When choosing therapy, it is also important to consider potential neurotoxicity. Interested readers are referred to comprehensive reviews on this topic [25, 26]. Briefly, 4–12% of children suffer a neurotoxic serious adverse event (SAE) such as seizures, stroke-like syndrome, posterior reversible encephalopathy syndrome, and/or long-term neurocognitive deficits [27]. ALL patients score 6–8 IQ points lower than controls [6] and 15–35% of children have significantly impaired working memory, attention span and/or executive functioning [28–30]. In addition, there are concerns that CNS-directed therapy may result in reduced cognitive reserve, thus risking early-onset dementia [9, 31–33]. The major culprit is thought to be methotrexate, although other agents may also contribute. Table 4 outlines the common agents used for ALL treatment, along with their CNS penetration and any known neurotoxic side-effects.

**Table 4:** CSF penetrance of systemically administered drugs commonly used in ALL treatment.

	CSF to plasma ratio, %	Established neurotoxic side effects
6-Mercaptopurine	26	N/A
Cyclophosphamide	20	N/A
Cytarabine	10–25	Cerebellar syndrome, seizures
Daunorubicin	ND	N/A
Dexamethasone	15	Neurobehavioural, pyschosis
Etoposide	ND	N/A
Fludarabine	Not known*	Encephalopathy, posterior reversible encephalopathy syndrome
Ifosfamide	38^†^	Encephalopathy
L-Asparaginase	ND^‡^	Cerebral venous sinus thrombosis
Methotrexate	3	Stroke-like symptoms, encephalopathy, seizure
Nelarabine	29^§^	Paraesthesia, weakness, seizure, cerebellar syndrome
Prednisolone	8	Neurobehavioural, pyschosis
Thiotepa	100	Heached, confusion, seizure, cerebellar syndrome, coma
Vincristine	5	Peripheral and autonomic neuropathy, posterior reversible encephalopathy syndrome, seizure

Median values used where possible. Table adapted from Balis et al. [36, 146–150].

*CSF* cerebrospinal fluid, *ND* not detected.

*Fludarabine is assumed to enter the CSF due to it’s neurotoxicity profile and case reports of single agent efficacy in CLL with CNS involvement [150].

^†^The active metabolite of ifosfamide, 4-hydroxy-ifosfamide, has a reported median CSF to plasma ratio of 307%. Significant variability is reported between patients, in the most recent study 5/17 patients had undetectable 4-hydroxy-ifosfamide CSF levels [148].

^‡^Although asparaginase is not detected in the CSF, asparagine depletion of the CSF does occur with systemic administration. The extent of depletion varies with asparaginase formulation [149].

^§^Data from non-human primates.

Whilst all modern protocols achieve low rates of CNS relapse, the optimal treatment regimen to maximize CNS control whilst minimizing toxicity is currently unknown. One consistent observation is that trials that intensify CNS-directed therapy often have reduced CNS relapse rates but an excess of later BM relapses. This results in no differences in EFS or OS, or sometimes worse OS (as bone marrow relapses may be more difficult to salvage). This has led to the concept of BM and CNS relapses being “competing events”. Poor risk ALL may relapse early (often “on treatment”) in the CNS, due to less chemotherapy exposure and immune surveillance in this compartment. If CNS-therapy is intensified this risk is reduced, but the same poor risk features make the patient at higher risk of BM relapse once intensive systemic treatment has finished.

### Standard therapy

The most intensively studied areas are around (i) choice of glucocorticoid, (ii) systemic methotrexate regimen, (iii) the use of single versus triple intrathecal therapy, and (iii) the safety of omitting radiotherapy.

### Glucocorticoids

Dexamethasone is considered to be the most effective oral glucocorticoid to treat CNS ALL. This is based on several randomized clinical trials comparing prednisolone and dexamethasone, including the collaborative study group l’Associazione Italiana di Ematologia e Oncologia Pediatrica – Berlin Frankfurt Münster’s (AIEOP-BFM) ALL2000 [34] and the UK Medical Research Council ALL97 trials [35]. In both trials those in the dexamethasone arm experienced a significantly lower isolated CNS (iCNS) relapse rate, although without an improvement in overall survival (OS). This is likely to reflect dexamethasone achieving higher concentrations in the CSF with a longer half-life compared to prednisolone [36]. It is worth noting however that other trials have failed to show a benefit for dexamethasone [22, 37]. The outcome difference in part reflects the dose relationships [38]. In terms of toxicity, dexamethasone is associated with worse acute neurobehavioral side-effects as well as increased rates of systemic toxicities such as avascular necrosis compared to prednisolone. The neurobehavioral side effects can in some patients be ameliorated by co-administration of hydrocortisone at physiological doses [39].

### Systemic methotrexate

Intravenous methotrexate regimen, and glucocorticoid choice, were the subjects of the Children’s Oncology Group’s (COG) AALL0232 trial [37]. 3154 high risk B-ALL patients aged 1–30 years were randomized to receive high-dose (5000 mg/m^2^) or Capizzi regimen (100 mg/m^2^ then escalating) intravenous methotrexate during interim maintenance. Am improvement in 5-year EFS was reported with the high-dose regimen. This was associated with a reduced, but non-significant, CNS relapse rate without excess toxicity. Converstly when studied in T-ALL, in COG AALL0434, the Capizzi regimen was superior to high-dose methotrexate by 5-year EFS and OS [40].

### Intrathecal therapy

Given the poor CSF penetration of many systemic anticancer agents (Table 4), direct injection of chemotherapy into CSF – so called intrathecal (IT) therapy – is an essential part of CNS-directed therapy. Although IT drug administration involving methotrexate, cytarabine, and/or glucocorticoids has been used for many decades, recent clinical trials have continued to investigate the optimal protocol.

COG AALL1131 hypothesized that prophylactic triple intrathecal therapy (TIT) (methotrexate, cytarabine and hydrocortisone) would improve EFS, due to reduced CNS relapses, compared with single intrathecal therapy (methotrexate) (IT MTX) post-induction [41]. The trial enrolled 1734 high-risk B-ALL patients aged 1–31 years, but CNS3 patients were excluded. The trial was stopped early after a futility boundary was crossed showing that TIT could not be superior to IT MTX. There was a (non-significant) trend towards reduced CNS relapse rates in the TIT arm but this was balanced by an opposite (also non-significant) trend for increased isolated BM relapses in this arm. A similar observation was seen in earlier COG trials for standard-risk ALL [42]. In both trials, there was no significant difference in overall rates of neurological toxicity between IT MTX and TIT.

### Radiotherapy

In the last decade many ALL trials have explored reducing or omitting the use of cranial irradiation and, therefore, the associated toxicities without sacrificing event-free survival. The consistent finding of these trials is that in the frontline setting cranial irradiation can be safely excluded in conjunction with an appropriately augmented systemic regimen. A meta-analysis, published in 2016, included over 16,000 patients, aged 1–18 years, enrolled across 10 study groups, and concluded that cranial irradiation does not impact CNS relapse risk in modern protocols [43]. This meta-analysis also included subgroup analysis for CNS3 patients. In this subgroup cranial irradiation was found to reduce the risk of isolated and combined CNS relapses, but neither EFS nor OS. Thus, in the majority of contemporary ALL protocols radiotherapy is reserved for relapsed disease, although some trial groups still use radiotherapy upfront for selected high-risk groups as well as part of conditioning prior to hematopoietic stem cell transplantation [44] (Table 2).

### Considerations for special groups

#### Philadelphia chromosome-positive ALL – choice of tyrosine kinase inhibitor

Following pre-clinical murine model data and case series data from Porkka et al. that demonstrated efficacy of dasatinib in CNS disease [45], COG AALL0622 [46] assessed the use of upfront dasatinib instead of cranial radiotherapy to prevent CNS relapse in Ph+ ALL. This single arm trial reported relatively high CNS relapse rates at 15% and made comparisons with historical controls. This strategy is complicated by differing rates of radiotherapy and hematopoietic stem cell transplant (HSCT) between the groups. Overall, the results suggest dasatinib alone (without cranial radiotherapy) may be insufficient to reduce CNS relapses rate in Ph+ ALL. A direct randomized comparison of imatinib versus dasatinib was performed by the Chinese Children’s Cancer Group study ALL-2015 in 189 pediatric patients with Ph+ ALL [47]. The number of patients with CNS disease at presentation was low (CNS3 *n* = 6) as was the total number of any CNS relapse events (*n* = 10). However, the investigators found a significantly reduced risk of iCNS relapse in the dasatinib arm, but no difference in any CNS relapse. This difference could potentially reflect the higher dasatinib-imatinib dose relation used compared to dosing currently used in other protocols (80 mg/m^2^ and 300 mg/m^2^ used in ALL-2015 versus 60 mg/m^2^ and 340 mg/m^2^ in COGAALL0622 and COGAALL0031 [46, 48], respectively), and the ongoing EsPhALL2017/COGAALL1631 protocol (ClinicalTrials.gov Identifier: NCT03007147).

#### T-ALL

T-ALL patients often receive intensified systemic and CNS-directed therapy. Many trial groups still routinely treat T-ALL patients with CNS radiotherapy, especially if they display other high-risk features such as hyperleukocytosis, although several groups have achieved good results despite omitting radiotherapy [49]. Nelarabine in frontline treatment was assessed in the randomized trial COG AALL0434; nelarabine versus no nelarabine in combination with intensive chemotherapy [50]. The nelarabine arm was found to significantly reduce the rates of isolated and combined CNS relapse although did not have a significant impact on OS. Other differences in systemic therapy may have also contributed to these results, such as less asparaginase in the non-nelarabine arm.

### Conclusion

Similar cure rates are achieved using a variety of regimens combining systemic multi-agent chemotherapy with effective CNS-directed therapy – no one approach is clearly superior. It is hoped that better biomarkers will enable evaluation of the true impact of different agents on clearance of CNS-ALL, but until then choice of therapy should aim at ensuring adequate targeting of the CNS compartment while minimizing short- and long-term neurotoxicity.

## Challenge 3 – What are the best approaches for CNS-relapse?

Evaluating the best treatment strategy for CNS relapse is even more challenging. Here small patient numbers, heterogeneity of frontline therapy, a lack of novel drugs and concerns regarding augmented neurotoxicity of immunotherapies have hampered progress.

Relapse of ALL is usually classified as either isolated bone marrow, isolated CNS (iCNS) or other extramedullary sites, or combined relapse involving two or more of these sites, most being BM + CNS. Even in the case of iCNS relapses submicroscopic marrow involvement is often seen [51], and it is also likely that subclinical CNS involvement is present in patients with an apparently isolated BM relapse. Thus, both systemic and CNS-directed therapy is essential for cure regardless of the site of relapse. Timing of relapse should also guide treatment choice. CNS relapses are usually categorized as ‘early’ or ‘late’. Two trials involving relapsed ALL, UK ALL R3 and IntReALL 2010, used the following definitions: very early relapse is those <18 months from diagnosis (and <6 months from completion of treatment in IntReALL 2010), early relapse as >18 months from diagnosis but <6 months from completion of treatment, and late relapse as those >6 months from completing treatment [52].

### Very early/Early CNS-relapses

The optimal therapy of iCNS relapse remains controversial. Early iCNS relapses have poor outcomes with EFS/OS rates of 41%/52% on COG AALL0433 and very similar outcomes from other groups [53]. Randomized trials comparing HSCT to chemotherapy have not been feasible, but because survival is <50%, many groups treat early iCNS relapses with intensive systemic chemotherapy followed by HSCT [54]. With small patient numbers, non-significant trends favoring HSCT over chemotherapy and cranial radiation were reported on COGAALL0433, UKALL R3, and single institution studies. A retrospective analysis of Italian children treated with HSCT for isolated extramedullary relapse from 1990 to 2015 showed improvements in 10-year survival rates; specifically survival in those with very early isolated extramedullary relapses was 56% with HSCT compared to historical rates of 20% to 30% with chemo-/radiotherapy only [55]. Early combined BM and CNS relapses also appear to benefit from HSCT. A trend towards a reduced rate of post-transplant CNS relapse but without OS benefit was found in a prospective trial involving cranial boost in the HSCT total body irradiation conditioning [56].

### Late CNS relapses

Historically, late iCNS relapses have had an excellent outcome on protocols using high dose chemotherapy and cranial radiotherapy of 24 Gy. In the UKALL R3 trial this group had a 5 yr EFS of 81% and OS of 85% [52]. Unfortunately, attempts to reduce radiotherapy doses or delay treatment to minimize toxicity in this good prognosis group have led to inferior outcomes [57]. Moreover, recent data suggest that the prognosis of late isolated extramedullary relapses may be worse than previously reported [58]. This may reflect use of more intensive first-line therapy, especially dexamethasone, which may have changed the biology of late relapses as well as selected out the most resistant patients. Clearly efficacious and low toxicity approaches are needed.

### The role of immunotherapy

Novel immunotherapies including bi-specific T cell engagers and antibody-drug conjugates have revolutionized the management of relapsed and refractory ALL in the last decade and are starting to be used for high-risk patients in front line settings. Evaluation of the CNS activity of these agents has been difficult as patients with CNS relapses were often excluded from early trials due to concerns of enhanced risk of neurotoxicity and the difficulties in measuring disease response in the CNS. Recently prospective Phase 3 studies from Europe [59] and the US [60] have examined the efficacy of Blinatumomab in released ALL. Overall, post-reinduction consolidation with blinatumomab in COG AALL1331 gave equivalent or better outcomes than chemotherapy alone and a comparatively favorable side effect profile [59, 60]. However, on subgroup analysis, this approach did not appear to be effective in those with isolated extramedullary (including iCNS) relapses [58]. This may reflect reduced penetration of Blinatumomab into extramedullary sites [61]. CAR-T cell therapy for CNS relapses remains experimental and is discussed under “better drugs” below.

### Novel agents

Despite use of all the agents described above there are a small but significant population of patients who present with recurrent or refractory CNS relapses. Clinical trials are hampered by the absence of sensitive biomarkers to monitor disease response similar to BM MRD. To this adds lack of access to precision medicine umbrella trials due to the challenges in obtaining material for genomic profiling and uncertainty regarding drug penetration into the CNS. As a result, iCNS relapses are excluded from current Phase I/II trials for relapsed ALL, and no randomized trials have addressed the optimal approach for this group. There are however isolated case reports and small case-series indicating efficacy of some novel treatment approaches including intrathecal rituximab, thiotepa and liposomal cytarabine (Depocyte) [62–64]. In addition, intrathecal etoposide is used in brain tumor patients and has antileukemic activity and may thus be another option [65].

### Potential solutions

Advances in treatment of CNS leukemia are hampered by two main issues. Firstly, the lack of sensitive and specific diagnostic, response, and prognostic biomarkers able to risk-stratify CNS-directed therapy and to measure the efficacy of novel treatment approaches. Secondly, the lack of non-toxic novel agents with activity against CNS leukemia. Recent progress in these two vital areas is discussed below.

## Solution 1 – Better biomarkers

**Fig. 1:**
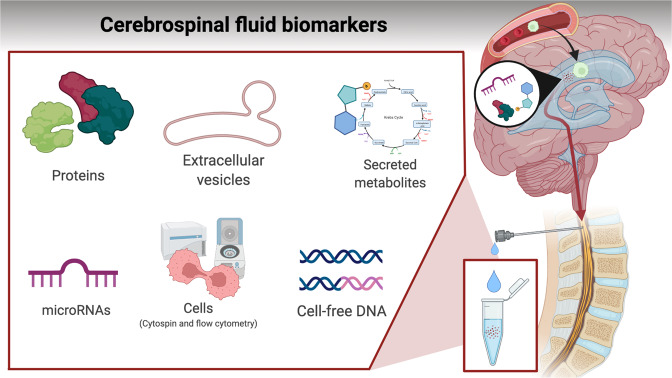
Soluble biomarkers secreted to cerebrospinal fluid or hypothesized to prime CNS compartments for transmigration of leukemic cells. Biomarkers may comprise leukemic-derived vesicles, secreted proteins and metabolites, or cell-free DNA.

The ability to accurately diagnose and quantify CNS ALL and its response to treatment will unlock the door to risk-adapted therapy as well as facilitate Phase I/II trials for novel CNS-active agents. Options include more sensitive and reliable detection of leukemic cells in CSF, or measuring soluble biomarkers secreted from leukemic cells in situ. Figure 1 and Table 5 outlines the different classes of biomarkers under investigation, which are discussed below.

**Table 5:** Cellular and soluble biomarkers associated with CNS leukemia [10, 24, 68–70, 74–78, 80, 83, 85, 86, 89, 151].

Biomarker(s)	Detection method	Study design	Patient cohort	Results
Leukemic cells				
Biomarkers related to hypoxic adaption [85]	Immunohistochemical analysis, gene set enrichment analysis, mass spectrometry and quantification of VEGFA transcripts with microarray	Experimental	CNS- and bone marrow-derived leukemic cells from xenografts recipients of pediatric ALL patients (*n* = 4)	B-ALL cells that infiltrate the CNS are more quiescent with less oxygen consumption but with greater glycolytic activity compared to those residing in bone marrow
Biomarkers related to metabolic plasticity [86]	Mass spectrometry (gas and liquid gas chromotography, qPCR, RNA sequencing)	Experimental	ALL live cells, CSF from normal controls and CSF from ALL patients	CNS leukemic blasts adapt metabolically to the CNS environment dependent on Stearoyl-CoA desaturase (SCD1). SCD1 overexpression in vivo increased CNS disease, whilst genetic or pharmacological inhibition of SCD1 descreased CNS load
Circulating leukemic blasts in CSF [151]	Cytospin with TdT immunostaining	Prospective non-intervention study	TdT+ ALL (*n* = 100), TdT+ acute nonlymphoblastic leukemia (ANLL) (*n* = 8) or TdT+ non-Hodgkin’s lymphoma (NHL) (*n* = 5). 1643 CSF samples were collected at diagnosis and during follow-up	CNS involvement in diagnostic and follow-up samples: 9.1% by TdT and 3.9% by cytospin. 74% of TdT+ samples were followed by overt CNS leukemia and two consecutive findings of TdT+ cells were always followed by overt CNS leukemia
Circulating leukemic blasts in CSF [68]	Flow cytometry (6-color)	Prospective non-intervention study	Newly diagnosed pediatric ALL (*n* = 108)	CNS involvement at diagnosis: 28% by flow cytometry vs 3% by cytospin. 3-year cumulative incidence of relapse: 10.7% for flow positive group vs 6.9% for flow negative group (*P* = 0.648)
Circulating leukemic blasts in CSF [69]	Flow cytometry (3–8 color)	Prospective non-intervention study	Newly diagnosed pediatric ALL (*n* = 214)	CNS involvement at diagnosis: 17% by flow cytometry vs 10% by cytospin. 5-year cumulative incidence of relapse: 29% for flow positive group vs 7% for flow negative group (*P* = 0.028)
Circulating leukemic blasts in CSF [70]	Flow cytometry (8-color)	Prospective non-intervention study	Newly diagnosed pediatric ALL (*n* = 87)	CNS involvement at diagnosis: 41% by flow cytometry vs 8% by cytospin. Relapse rate: 11.8% for flow positive group vs 4.0% for flow negative group (*P* = 0.15)
Circulating leukemic blasts in CSF [24]	Flow cytometry (6-color)	Prospective non-intervention study	Newly diagnosed pediatric ALL (*n* = 673)	CNS involvement at diagnosis: 25% by flow cytometry vs 13% by cytospin. 4-year cumulative incidence of relapse: 16.5% for flow positive group vs 5.6% for flow negative group (*P* = 0.0013). Cytospin and/or flow positivity was an independent risk factor for relapse in multivariate analysis (HR: 2.2, *P* = 0.042)
Circulating leukemic blasts in CSF [10]	Flow cytometry (6-color)	Prospective non-intervention study	Newly diagnosed pediatric ALL (*n* = 255)	CNS involvement at diagnosis: 23% by flow cytometry vs 51% by cytospin. High CNS2 rate potentially due to false-positives (cytospin evaluated by four observers in two different laboratories and defined as positive if one of the observers identifies 1 leukemic blast). 5-year RFS for CNS2 patients (*n* = 107): 87.9% for flow positive group vs 100% for flow negative group (*P* = 0.003)
DNA from CSF cells [76]	PCR for VDJ rearrangements	Prospective non-intervention study	Newly diagnosed pediatric ALL without TLP (*n* = 37)	CNS involvement at diagnosis: 46% by PCR vs 5% by cytospin. 4-year EFS: 41% for PCR positive group and 84% for PCR negative group (*P* = 0.01)
DNA from CSF cells [74]	PCR for VDJ rearrangements	Prospective non-intervention study	Newly diagnosed pediatric ALL (*n* = 30)	CNS involvement at diagnosis: 20% by PCR vs 17% by cytospin. PCR confirmed confirmed cytospin results in 2 patients. Cytospin results could not be confirmed by PCR in 3 patients
DNA from CSF cells [77]	PCR for VDJ rearrangements	Prospective non-intervention study	Newly diagnosed pediatric ALL without TLP (*n* = 65)	CNS involvement at diagnosis: 47% by PCR vs 5% by cytospin. 5-year EFS: 54.8% for PCR and/or cytospin positive group vs 77.7% for PCR and cytospin negative group (*P* = 0.03). PCR and/or cytospin positivity not independent prognostic factor in mutivariate analysis (*P* = 0.15)
DNA from CSF cells [75]	PCR for VDJ rearrangements	Prospective non-intervention study	Newly diagnosed pediatric ALL (*n* = 38)	CNS involvement at diagnosis: 40% by PCR vs 13% by cytospin. PCR cofirmed cytospin results in 4 patients. Cytospin results could not be confirmed by PCR in 1 patient
Soluble biomarkers				
Cerebrospinal fluid proteome during PEG-asparaginase treatment [78]	Quantitative label-free LC-MS/MS (tryptic digest)	Cross-sectional observation	Newly diagnosed B- and T-ALL (*n* = 4) and lymphoblastic lymphoma (*n* = 1)	Proteome profiling of CSF altertions during PEG-asparaginase treatment. 635 proteins (406 proteins with 2 or more peptides), here 35 protens had significantly altered intensities throughout day 0, day 8 and day 29 (*P* < 0.05 following Benjamini-Hochberg correction)
Cerebrospinal fluid proteome alterions comparing before vs after induction therapy [80]	Quantitative label-free LC-MS/MS (tryptic digest)	Cross-sectional observation	Pediatric patients with confirmed CNS B-ALL (*n* = 6)	428 proteins identified comparing pre vs post induction chemotherapy, here with 10 proteins being signficantly altered (*P* < 0.05 in a paired *t*-test without correction of multiple comparisons)
Extracellular vesicles (leukemia-derived exosomes for CSF-barrier transmigration) [83]	Fluorescent microscopy	Experimental	In vitro model with human malignant choroids plexus papilloma cell line as blood-CSF-barrier and three ALL cell lines (SD-1, Nalm-6, P12-Ichikawa)	Leukemia-derived exosomes may facilitate CNS invasion of ALL cell lines across the blod-CSF-barrer witout destruction of the barrier integrity
microRNA [89]	qPCR	Cross-sectional observation	Pediatric ALL and AML patients. Discovery cohort using CSF with CNS-ALL (*n* = 4) and CNS-naive ALL (*n* = 4) matched patients. Validation cohort using CSF with ALL, AML og mixed phenotype patients, including CNS-ALL (*n* = 11) and CNS-naive (*n* = 13) patients	In total, 47 candiate microRNAs were selected for profiling. Based on the discovery cohort, miRNA-181a was overexpressed 52-fold in CNS-ALL patients in the validation cohort

*ALL* acute lymphoblastic leukemia, *CNS* central nervous system, *CSF* cerebrospinal fluid, *TdT* terminal deoxynucleotidyl transferase.

### Improving detection of cells in CSF

Recently, multicolor flow cytometric analysis of CSF has been applied as a more sensitive method for detection of leukemic blasts in the CSF compared to cytospin. In flow cytometry, blasts are identified based on their aberrant expression of leukemia-associated immunophenotype markers, making this technique highly specific [66]. Furthermore, flow cytometry can rapidly and precisely quantify the expression of multiple cell surface molecules even when the cell count is 1000 fold lower than the upper normal limit of leukocytes in CSF (5 × 10^9^/L) leading to a much higher sensitivity than conventional cytology [66]. Furthermore, the cells can be fixated prior to flow cytometry, e.g. in specialized CSF Transfix® tubes, which preserves the cells and allows for delaying analysis of CSF for 48–72 h, thus facilitating centralized analysis [67].

At diagnosis, CNS involvement has been detected by CSF flow cytometry in 17–41% of cases compared to only 3–10% of cases classified as CNS2 and CNS3 by cytospin in children with ALL [24, 68–70]. One study also investigated the clearance of leukemic blasts during induction therapy and found that 7.5% of flow positive patients at diagnosis remained positive at day 15 [24]. It was recently shown in a large study (*n* = 673) by the NOPHO group that CSF flow positivity at diagnosis was an independent risk factor for relapse among children and adolescents with ALL [24]. This association was confirmed in a study by COACG group in patients with low level CNS disease (classified as CNS2 by cytospin) [10]. Two other studies also showed that relapse occurred more frequently among patients who were CSF flow positive at diagnosis, but results did not reach statistical significance, likely due to the small study cohorts [68, 70].

TLP with blasts (TLP+) has previously been associated with increased risk of CNS relapse in childhood ALL [8, 71–73]. In the aforementioned NOPHO study, TLP at diagnosis was only associated with a higher risk of relapse in patients, where presence of blasts were confirmed by flow cytometry [24]. Accordingly, the current European ALLTogether1 treatment protocol (NCT04307576) requires flow cytometry confirmation of CNS involvement in case of TLP. The prognostic significance of the patient’s blast level at diagnosis and rate of clearance of leukemic blasts during treatment is also being tested in the ALLTogether1 trial to determine if these parameters can be used to assign a CNS-relapse score on which to base a future randomized trial of risk-adapted CNS-directed therapy to balance treatment efficacy and toxicity.

Another technique that has been proposed for detection of submicroscopic levels of CNS involvement is PCR on cell-free DNA in CSF. PCR on CSF DNA is typically performed with the patient-specific primers against variable regions in immunoglobulins and the T-cell receptor generated for bone marrow MRD. In pediatric ALL patients, PCR detected CNS dissemination in 20–47% of cases compared to 5–17% by cytospin [74–77]. The rates of CNS involvement at diagnosis by PCR are equivalent to the rates obtained by CSF flow cytometry. However, in several studies it was not possible to analyze a large fraction of the CSF samples by PCR due to poor quality of the DNA or lack of suitable primers [75–77]. In studies sampling CSF during treatment, patients quickly became negative by PCR [74, 75, 77], which suggest that more sensitive techniques, such as flow cytometry or next generation sequencing, are needed to assess treatment response in the CNS.

### Soluble biomarkers for CNS-ALL

Given that leukemic cells in the CNS are often adherent to stroma rather than free-floating in CSF it is hypothesized that measurement of a CSF biomarker that is released or taken-up by leukemic cells may provide a more relevant and quantifiable measure of disease burden than cell-based methods. Biomarkers may reflect a variety of biological mechanisms, such as transmigration and adhesion to CNS-stroma, metabolic plasticity, and cellular cross-talk. It may, however, prove difficult to reduce this complexity into single biomarkers, and combinatorial risk-scores incorporating multiple biomarkers and/or genetic/demographic features may be needed.

Currently, evidence from mass spectrometry-based proteomics of CSF is sparse, with only a few small cohort studies published. However, characterization of alterations in the CSF proteome and/or metabolome may convey measures of CNS malignancy. Here, aberrant catalase levels have been reported in the CSF proteome of B- and T-ALL patients with cytospin CNS2-status [78]. Catalase occurs in aerobically respiring cells and promotes growth of leukemic cells [79], which may support CNS-ALL blasts adapting to hypoxic glycolysis. Further, the serine protease kallikrein-6 seems specifically upregulated in the CSF-proteome of CNS-ALL patients [80]. The enzyme degrades extracellular matrix and facilitates local tumor invasion and infiltration [81], and could therefore indicate mechanisms involved in CNS infiltration. Finally, specific alterations in CSF correlate with rare cases of cancer prone syndromes, e.g. an ATP-dependent RNA helicase (DDX41), which has been detected in the CSF proteome of some ALL patients [78].

Recently, blast-derived extracellular vesicles have been hypothesized to foster malignant transformation of leukemic cells and facilitate transmigration across the blood-CSF-barrier by priming of choroid plexus cells [82, 83]. Technological progress has enabled fluorescent labeling of extracellular vesicles [84] may also provide future biomarkers for CNS invasion and risk of CNS relapse.

Metabolic plasticity may be pivotal for adapting to the low nutrient microenvironment in CSF, and metabolites and metabolic regulators are potential biomarkers for CNS-ALL. Distinct biological mechanisms have been described, including hypoxic adaptation by upregulation of vascular endothelial growth factor A (VEGFA) [85] and metabolic adaption by Stearoyl CoA desaturase (SCD1) dependent fatty-acid synthesis [86]. Although rather non-specific, lactate dehydrogenase (LDH) has been used a biomarker for CNS lymphoma diagnostics, and elevated LDH levels may also indicate CNS involvement in ALL [87].

Circulating microRNA (miRNA) in CSF have been linked to CNS involvement. Many miRNAs have been reported, yet not validated, but may include high expression of the miRNA-181-family, miR-34a, miR-128a, miR-128b, and miR-146a, in CSF positive relative to CSF negative patients [88, 89].

Finally, cfDNA is released from cells into the surrounding body fluids by a variety of mechanisms including active release and secondary to apoptosis and cell turnover. Measurement of cfDNA in plasma (and/or CSF for brain tumors [90]) detects solid tumors at early stages [91] and dynamically tracks treatment responses with levels rising prior to overt disease recurrence [92]. ALL is a fast-growing malignancy with rapid cell turnover, and CNS blasts reside directly within the CSF compartment. Therefore, CSF cfDNA would be expected to provide a sensitive and specific biomarker for detecting and tracking CNS involvement. However, evidence for this is currently lacking.

## Solution 2 – Better drugs

**Fig. 2:**
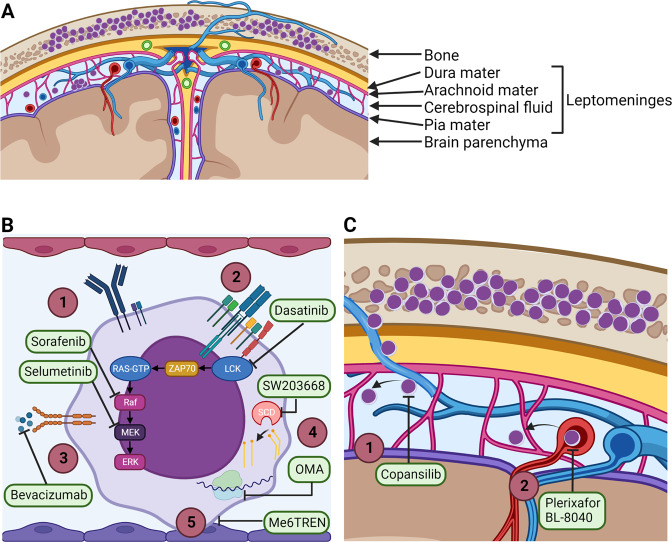
Mechanisms of action of drugs that target leukemic cells within the CNS. **A** Coronal section of human brain showing the meninges and the meningeal vasculature. The leptomeninges consist of the arachnoid mater, the pia mater and the subarachnoid space. The subarachnoid space is filled with CSF, veins, arteries and arachnoid trabeculae extending from the arachnoid mater to the pia mater. **B** Novel drugs that target survival mechanisms employed by leukemia cells in the leptomeninges [1]. Sorafenib and selumetinib inhibit Ras/Raf/MEK/ERK signaling downstream of B-cell receptor activation [2] Dasatinib inhibit LCK signaling downstream of T-cell receptor activation [3]. Bevacizumab sequesters VEGF-A and inhibit binding to the VEGFR2 [4]. SW103668 inhibit SCD-mediated enzymatic conversion of saturated fatty acids to mono-unsaturated fatty acids and OMA inhibit ribosome mRNA translation [5]. Me6TREN inhibit adhesion of leukemia cells to meningeal cells. **C** Novel drugs that target invasion mechanisms employed by leukemia cells during dissemination to the leptomeninges [1]. Copansilib inhibit integrin α6-mediated migration of leukemia cells along emissary vessels [2]. Plerixafor or BL-8040 block CRCX4-mediated migration across meningeal blood vessels. LCK lymphocyte specific cell-kinase, CNS central nervous system, CSF cerebrospinal fluid, SCD stearoyl-CoA desaturase, VEGF vascular endothelial factor, OMA omacetaxine mepesuccinate.

Real advances in treating CNS leukaemia will require new therapeutics. Replacement of conventional agents such as methotrexate with less neurotoxic alternatives will benefit all patients. Novel agents are also needed to unlock the potential of biomarker driven approaches to identify high-risk patients and for those with refractory disease. An overview of agents in preclinical development and clinical testing are shown in Fig. 2 and Table 6.

**Table 6:** Overview of clinical and preclinical evidence for anti-CNS leukemia effects of novel therapeutic agents [85, 86, 93, 95, 96, 100, 103, 105–108, 111, 113, 115, 119, 121, 124].

Drug name	Target	Study design	Patient cohort/preclinical model	Results
Immunotherapies				
Tisagenlecleucel (CTL019) [93]	CD19	Phase I–II clinical trial (case report of 2 enrolled patients)	Pediatric relapsed or refractory BCP-ALL (*n* = 2)	Complete response achieved in both patients. 1 patient relapsed after 2 months, 1 patient still relapse-free 11 months after treatment. CAR-T cells observed in bone marrow and CSF.
Anti-CD19 CAR-T cells [100]	CD19	Phase I–II clinical trial (case report of 3 enrolled patients)	Adult relapsed or refractory B-ALL with CNS involvement (*n* = 3)	Complete response achieved in all patients with relapse-free survival of 2–5 months. CAR-T cells observed in CSF, but dynamics varied between patients.
Anti-CD19 CAR-T cells containing co-stimulator CD28 or 4-1BB [95]	CD19	Phase I–II clinical trial (case report of 1 enrolled patient)	Pediatric relapsed or refractory BCP-ALL with CNS involvement (*n* = 1)	Complete response achieved with 36 months of relapse-free survival.
Tisagenlecleucel (CTL019) & huCART19 [96]	CD19	Post-hoc analysis of 5 clinical trials	Pediatric or adult relapsed or refractory B-ALL (*n* = 195)	Patients categorised as CNS pos or neg based on CNS status at relapse or within the 12 months preceding CAR T-cell infusion. No significant difference in complete response rates (64% vs 66%) or 2-year overall survival (83% vs 71%) for CNS pos vs CNS neg patients.
Survival				
Sorafenib [105]	Raf	Phase II clinical trial	Adult refractory CNS ALL (*n* = 26)	Complete response achieved in 17 patients and a partial response achieved in two patients. 2-year event free survival of 75% and 2-year overall survival of 76.9%.
Selumetinib(NCT03705507)	MEK	Phase I–II clinical trial	Pediatric and adult relapsed or refractory B- or T-ALL with RAS-pathway activating mutations (*n* = 42)	Ongoing clinical trial - no reported results yet. The aim is to investigate safety and efficacy of selumetinib in combination with dexamethasone.
U0126 [103]	MEK	Preclinical	Cell line ALL xenografts	Monotherapy significantly reduced leukemic load in CNS.
Selumetinib [106]	MEK	Preclinical	Patient-derived BCP-ALL xenografts	Monotherapy significantly reduced leukemic load in CNS.
Selumetinib [107]	MEK	Preclinical	Patient-derived BCP-ALL xenografts	Monotherapy significantly reduced leukemic load in CNS. Combination therapy with dexamethasone resulted in no detectable CNS leukemia in 2 of 3 xenografts.
Dasatinib [108]	LCK	Preclinical	Patient-derived T-ALL xenografts	Monotherapy significantly reduced leukemic load in CNS in 4 of 8 of xenografts. Combination therapy with dexamethasone resulted in no detectable CNS leukemia in more than 5 of 9 xenografts and significant reduction in leukemic load in CNS in remaining 4 xenografts.
Bevacizumab [85]	VEGF	Preclinical	Patient-derived BCP-ALL xenografts	Monotherapy significantly reduced leukemic load in CNS.
Bevacizumab [111]	VEGF	Preclinical	Patient-derived BCP-ALL xenografts	Monotherapy significantly reduced leukemic load in CNS.
SW203668 [86]	SCD	Preclinical	Patient-derived and cell line ALL xenografts	Monotherapy significantly reduced leukemic load in CNS.
Me6TREN [115]	Meningeal adhesion of leukemic cells	Preclinical	Patient-derived and cell line B-ALL xenografts	Combination therapy with cytarabine significantly reduced leukemic load in CNS compared to monotherapy with cytarabine.
Omacetaxine mepesuccinate [113]	Ribosome	Preclinical	Patient-derived B-ALL xenografts	Monotherapy significantly reduced leukemic load in CNS.
Invasion				
Copanlisib(NCT04803123)	PI3K	Phase I clinical trial	Adult relapsed or refractory B-ALL (*n* = 10)	Ongoing clinical trial - no reported results yet. The aim is to evaluate biologic markers of leukemia cell response following a single dose of copanlisib.
GS-649443 [119]	PI3K	Preclinical	Cell line BCP-ALL xenografts	Monotherapy significantly reduced leukemic load in CNS.
GS-649443 & Copanlisib [121]	PI3K	Preclinical	Cell line BCP-ALL xenografts	Combination therapy with GS-649443 and cytarabine significantly reduced leukemic load in CNS compared to monotherapy with cytarabine. Monotherapy with copanlisib significantly reduced leukemic load in CNS.
BL-8040(NCT02763384)	CXCR4	Phase II clinical trial	Adult relapsed or refractory T-ALL and T-LBL (*n* = 20)	Ongoing clinical trial - no reported results yet. The aim is to investigate safety and efficacy of BL-8040 in combination with nelarabine.
Plerixafor (AMD-3100) [124]	CXCR4	Preclinical	Patient-derived and cell line T-ALL xenografts	Monotherapy significantly reduced leukemic load in CNS.

*ALL* acute lymphoblastic leukemia, *BCP* B-cell precursor, *CAR-T* chimeric antigen receptor T-cell, *CNS* central nervous system, *CSF* cerebrospinal fluid.

### Novel immunotherapies

Chimeric antigen receptor T (CAR-T) cells targeting CD19 on B-ALL have demonstrated convincing evidence of activity in the CNS. In early-stage clinical trials, investigators have reported the presence of CAR-T cells within the CSF of treated ALL patients [93]. Subsequently, multiple case reports and case series have demonstrated clearance of CNS leukemia with CD19-targeted CAR-T cell treatment including those with iCNS disease [94, 95]. Post-hoc analysis from five CAR-T cell clinical trials with 195 patients with relapsed or refractory B-ALL, of whom 54% had evidence of CNS disease (usually as part of combined CNS and BM relapses), found similar rates of complete response and relapse-free survival irrespective of CNS status at relapse [96]. This conclusion was echoed by the Pediatric Real World CAR Consortium where they reported CD19 CAR-T therapy outcomes and toxcities for patients with CNS disease were similar to those with BM only involvement [97]. There are, however, reports of less favourable outcomes; a recently published international retrospective analysis reported a high rate of subsequent CNS relapse in those with iCNS disease (6 of 8 patients) following CD19 CAR-T therapy [61]. Although CAR-T cell therapy holds significant promise, the outcome data is somewhat inconsistent and longer follow-up is needed to determine the longevity of responses [61]. Emerging data from B-cell lymphoma patients suggests that CAR-T cells may undergo some degree of exhaustion or anergy in the CNS microenvironment leading to antigen positive relapses despite CAR-T cell persistence in the CSF [98]. One potential strategy for overcoming this challenge could be by repeated administration of CAR-T cells. Furthermore, CAR-T cell therapy may be associated with serious neurotoxicity in patients with high-burden CNS leukemia, however recent reports indicate that these toxicities are usually reversible with intensive supportive care [99, 100].

### Novel drugs that target cell survival mechanisms

One strategy is to target the leukemic cells that have already entered the CNS by disrupting the molecular mechanisms that support their survival in the CNS microenvironment. In the leptomeninges the leukemic cells are in direct contact with the CSF that has low oxygen and nutrient levels compared to the blood [101]. Recent evidence supports that B-cell receptor signaling through the Ras/Raf/MEK/extracellular signal-regulated kinase (ERK) pathway promotes survival of leukemic cells in the CNS [102–104]. In a phase II study, theatment with the Raf inhibitor sorafenib showed efficacy in patients with refractory CNS leukemia [105]. Preclinical xenograft studies have demonstrated inhibition of CNS leukemic load of the MEK inhibitor selumetinib alone [106] or in combination with dexamethasone [107]. In the SeluDex trial (NCT03705507), efficacy of selumetinib in combination with dexamethasone is being evaluated separately in the bone marrow and CNS in pediatric and adult patients with relapsed or refractory ALL, but no results have been reported yet. In T-cell ALL, inhibition of lymphocyte specific cell-kinase (LCK) (acting downstream of the T-cell receptor) by dasatinib has been shown to reduce cell proliferation in vitro and reduce leukemic load in the CNS [108]. Both Dasatinib and the Bcl-2 inhibitor Venetoclax penetrates the blood-brain-barrier and could be useful for CNS-involving T-cell ALL [109].

Two interesting therapeutic strategies that have not yet progressed to clinical testing, include disruption of metabolic adaption to the CNS microenvironment and contact-induced quiescence. Accumulating evidence supports that the low oxygen and nutrient availability in the CSF induce metabolic adaptations in the leukemic cells that promote their survival in the CNS microenvironment [110]. Several studies have shown that hypoxia-gene expression signatures are upregulated in leukemic cells located in the CNS, and that treatment with the anti-VEGF antibody bevacizumab significantly reduced CNS leukemia in ALL xenograft models [85, 111]. Fatty acid synthesis is also upregulated in ALL cells isolated from the CNS [86, 112], and pharmacological inhibition of the enzyme stearoyl-CoA desaturase (SCD) (converting saturated fatty acids to mono-unsaturated fatty acids) significantly reduced CNS involvement in a recent study [86]. A recent xenograft study not only confirmed the altered metabolic state of ALL cells within the CNS, but also identified mRNA translation as potential therapeutic target [113]. Treatment with the ribosome A inhibitor omacetaxine mepesuccinate reduced mRNA translation in patient-derived xenografts and reduced leukemic burden in the CNS [113]. Adhesion of leukemic cells to meningeal cells induces a dormant phenotype and increased resistance to chemotherapy in animal and cell line studies [114, 115]. The small molecule inhibitor Tris[2-(dimethylamino)ethyl]amine] (Me6TREN) in combination with cytarabine significantly inhibited CNS dissemination compared to monotherapy with cytarabine in xenograft models [115]. These studies highlight SCD, VEGF, mRNA translation and contact-induced quiescence as potential targets for CNS-directed therapy, but this needs to be explored in clinical trials.

### Novel drugs that target CNS invasion

Leukemic cells predominantly reside within the meninges that cover the surface of the brain and spinal cord and only invade the brain parenchyma in late-stage disease [116]. Due to the localization of leukemic cells, the leading hypothesis has been that the leukemic cells enter the CNS by crossing the blood-leptomeningeal-barrier in the meningeal microvessels or the blood-CSF-barrier at the choroid plexuses [117, 118]. However, recently leukemic cells were observed to migrate directly from the bone marrow and into the leptomeninges along the outer surface of vessels passing through fenestrations in the vertebral or calvarial bones [119]. Integrins α6 and α5 play a role in CNS involvement [120]. Treatment with PI3K inhibitors reduced expression of integrin α6 by cultured BCP-ALL cells, and reduced CNS involvement and prolonged survival in BCP-ALL xenografts [119, 121]. A phase I clinical trial with the pan-PI3K inhibitor copanlisib is currently ongoing to study the effect on α6 expression and lymphoblast proliferation in adult patients with refractory or relapsed B-ALL (NCT04803123). Various other integrins and adhesion molecules has been associated with CNS involvement in childhood ALL [117, 118, 122], but no drugs targeting these integrins or downstream signaling pathways have progressed to clinical testing yet.

Normal lymphocytes express numerous chemokine receptors, including CXCR3, CXCR4 and CCR7, which promote migration into the CNS through expression of the corresponding ligands in the brain endothelium and choroid plexus epithelium [123]. In T-ALL, CXCR4 expression in diagnostic bone marrow samples have been associated with CNS involvement [103] and treatment with the CXCR4 inhibitor plerixafor (AMD-3100) reduced CNS infiltration in T-ALL xenografts [124]. A phase II clinical trial is currently ongoing where the CRCX4 antagonist BL-8040 is added to nelarabine treatment in adult patients with refractory or relapsed T-ALL (NCT02763384). The results are awaited.

Since a large proportion of children with ALL already have CNS involvement at diagnosis, drugs blocking the entry of leukemic cells into the CNS may have limited effect in the clinical setting. Currently, it is not known if continuous trafficking of leukemic cells from the blood or bone marrow into the CNS occurs after initial CNS seeding. The therapeutic benefit of targeting CNS invasion in childhood ALL thus needs to be confirmed in clinical studies and carefully designed xenograft studies where treatment is initiated only after CNS involvement has been established.

Finally, one other barrier to effective new therapies is whether the drug can enter the CSF compartment. Intrathecal delivery circumvents the need to cross the blood brain/blood-CSF barrier, but it is onerous for families and repeated general anesthesia in children may provide an added neurocognitive burden [125]. Oral small-molecule inhibitors vary in their ability to enter the CNS compartment and drug-engineering may be needed to enhance CSF-penetrance of promising compounds [126, 127]. Alternatively, implanted intrathecal drug delivery devices may also be appropriate – especially in the CNS-relapse setting [128].

## Summary/conclusions

Optimizing treatment of the CNS remains a challenge in childhood ALL. Current approaches are intensive, non-discriminative, and cause significant morbidity, whilst treatment options for patients with relapsed/refractory CNS-ALL are limited. Solutions lie in new drugs and better biomarkers. The lack of accurate biomarkers is most critical. Without a means to measure treatment response the testing of novel agents becomes difficult. Moreover, biomarkers will help identify patients at low or high risk for CNS relapse. For low-risk children who currently receive large amounts of CNS-directed therapy, reducing this treatment burden would have significant health and economic benefits. For high-risk children novel approaches are needed, facilitated by new drugs. Fortunately, research into CNS-ALL has increased over the last decade and is starting to provide a better understanding of disease biology as well as putative drug targets and biomarkers. Some biomarkers, such as CSF-flow cytometry, are now being tested in prospective trials. Novel drugs are also being tested in Phase I/II trials, although wider access for iCNS relapse patients is needed. The future is hopeful for improved management of the CNS over the next decade.
